# Editorial for the Special Issue on Laser Additive Manufacturing: Design, Processes, Materials and Applications

**DOI:** 10.3390/mi13122057

**Published:** 2022-11-24

**Authors:** Jie Yin, Yang Liu, Ping Zhao

**Affiliations:** 1 Gemological Institute, China University of Geosciences, Wuhan 430074, China; 2 Advanced Manufacturing Research Institute, China University of Geosciences, Wuhan 430074, China; 3 Faculty of Mechanical Engineering & Mechanics, Ningbo University, Ningbo 315211, China; 4 Department of Microtechnology and Nanoscience, Chalmers University of Technology, 41296 Gothenburg, Sweden

Laser-based additive manufacturing (LAM) is a revolutionary advanced digital manufacturing technology developed in recent decades, which is also a key strategic technology for technological innovation and industrial sustainability. This technology unlocks the design and constraints of traditional manufacturing and meets the needs of complex geometry fabrication and high-performance part fabrication. A deeper understanding of the design, materials, processes, structures, properties and applications is desired to produce novel functional devices, as well as defect-free structurally sound and reliable LAM parts.

The topics in this Special Issue include macro- and micro-scale additive manufacturing with lasers, such as structure/material design, fabrication, modeling and simulation, in situ characterization of additive manufacturing processes and ex situ materials characterization and performance, with an overview that covers various applications in aerospace, biomedicine, optics and energy.

In this Special Issue, papers on different subjects were published after the high-quality reviewing process, with a total of 16 contributions (14 original research papers and 2 review papers) and times of viewed over 13K (as of 15 November 2022). Six articles were selected as Editor’s Choice and one article was selected as the Issue Cover of Volume 13, Issue 8 (https://www.mdpi.com/2072-666X/13/8 (accessed on 25 August 2022)). Each of them is briefly introduced below according to four aspects (design, processes, materials and applications) of laser additive manufacturing in this Special Issue.

## 1. Design of Laser Additive Manufacturing in This Special Issue

The design of laser additive manufacturing covered in this Special Issue includes the structural design (e.g., the negative Poisson’s ratio endovascular stents [1], the multi-material heterostructures [2]) and the material design (e.g., the in situ synthesis of a novel alloy [3] and composite modification [4]).

Chen et al. [1] reported that anti-tetrachiral auxetic stents with negative Poisson’s ratios (NPR) were designed and fabricated via LAM. The cytocompatibility tests indicate the envisaged cell viability and adhesion of the vascular endothelial cell on the LAM-fabricated anti-tetrachiral auxetic stents. This study is a first step toward the structural design and manufacturing of endovascular stents with negative Poisson’s ratios, which exhibit predestined biocompatibility.

Song et al. [2] studied the microstructure, element diffusion, microhardness changes and phases at the interface of NiTi/CuSn10 multi-material heterostructures. They found that the formation of the N4Ti3 strengthening phase improves the microhardness of the interface, making the microhardness of the interface significantly higher than that of both sides. Under the optimized process parameters, the maximum strength can reach 309.7 ± 34.4 MPa.

Yang et al. [3] reported that a supersaturated solid solution of a Mg–Zn alloy with good cytocompatibility was achieved using mechanical alloying (MA) combined with laser sintering. Note that the supersaturated solid solution Mg–Zn powders were firstly prepared using MA, which can break through the limit of phase diagram under the action of forced mechanical impact.

Zheng et al. [4] reported that polyamide 12 (PA12) and carbon-fiber-reinforced polyamide 12 (CF/PA12) composites were fabricated using selective laser sintering (SLS) LAM. They investigated the coupling effects of the strain rate and hygroscopicity on the compressive mechanical properties. Results showed that the CF/PA12 had a shorter saturation time and lower saturated water absorption under the same conditions, indicating that the SLS of CF/PA12 had lower hydrophilia and higher water resistance when compared to the SLS of PA12. This work provided a basic knowledge of the mechanical properties of SLS polyamide under different load and saturated-water conditions and, thus, is helpful to widen the application of SLS PA products in harsh environments.

## 2. Processes of Laser Additive Manufacturing in This Special Issue

This Special Issue article focuses on continuous-wave (CW) laser additive manufacturing (e.g., laser powder bed fusion (L-PBF), laser direct energy deposition (L-DED)), but also includes pulsed-wave (PW) laser advanced manufacturing (e.g., femtosecond-pulsed laser machining, two-photon polymerization [5]) and heat treatment annealing processes [6]. It is worth mentioning that laser additive composite manufacturing (e.g., CW L-PBF & CW L-DED [7]), laser subtractive composite manufacturing (CW laser and PW femtosecond laser machining [8]) and laser additive & subtractive composite manufacturing (CW L-PBF and PW femtosecond laser surface ablation modification [9]) are also current research frontiers in laser advanced manufacturing.

Zhang et al. [7] established an analytical model to predict the layer-dependent processing parameters for fabricating 07Cr15Ni5 steel via L-DED on a CuCr substrate. Changes in the effective thermal conductivity and specific heat capacity with the layer number, as well as the absorption rate and catchment efficiency with the processing parameters, are considered. The parameter maps predicted by the model are in good agreement with the experimental results.

Kang et al. [8] proposed a hybrid dissection method, where a high-power CW laser was firstly employed to make a tapered groove on the shell’s surface and then a femtosecond-pulse laser was used to micromachine the groove in order to obtain a cutting kerf. By using the hybrid method, the cutting efficiency was improved about 49-times compared to the femtosecond laser cutting. This method provides a high-efficiency and non-thermal cutting technique for reclaimed metallic neutron tube shells with millimeter-level-thick walls, which has the advantages of non-contact, minimal thermal diffusion and being free of molten slag.

Meng et al. [9] reported that L-PBFed 17-4PH SS was treated by a femtosecond laser to regulate the surface structures and corrosion-resistance behaviors. The Cr, Cu and other alloying elements precipitated on the laser-ablated surface were beneficial to the formation of a passivation film, leading to an improved corrosion-resistance performance.

Wang et al. [10] reported a review of the macroscopic and microstructural features of metallic coating created by pulse-wave (PW) laser material deposition (LMD). The research status of temperature field simulation, surface quality and microstructural features, including microstructures, microhardness, residual stress and cracking, as well as corrosion behavior of metallic coating created by pulsed-laser material deposition, were reviewed.

## 3. Materials of Laser Additive Manufacturing in This Special Issue

This Special Issue article covers a wide range of forming materials, including steel [1,9] and other iron-based alloys [11], magnesium alloys [3], titanium alloys [6,12], copper alloys [7], aluminum alloys [13], composites (e.g., CF/PA12 [4], copper/titanium-coated diamond [14]) and, in particular, metallic multi-materials (e.g., NiTi/CuSn10 [2], copper/steel [7]) and 4D-printing materials (e.g., NiTi shape memory alloys [2,15], smart materials for soft interactive hydrogel [5]).

Cui et al. [12] investigated three types of Ti-6Al-4V (TC4) materials with different porosities fabricated via L-PBF using different printing parameters. Experimental results indicate that the porosity significantly affects their dynamic response (the yield strength and spall behaviors).

Xu et al. [13] reported that W-particle-reinforced Al alloys were prepared on a 7075-aluminum alloy surface via laser-melt injection (LMJ) to improve their wear resistance. Results confirmed that a W/Al laser-melting layer of about 1.5 mm thickness contained W particles and Al4W was formed on the surface of the Al alloys.

Zhang et al. [14] used the L-PBF technique to fabricate titanium- and copper-coated diamond/copper composites. The microstructure, roughness, interface bonding, thermal and mechanical performance were studied. The work offered electroless plating and evaporation methods for L-PBF to coat copper and titanium on the diamond particle surface for improving the wettability of the diamond/copper interface, which opened up a new method for laser 3D-printing technology to print a broad range of diamond-particle-reinforced metal matrix composites.

Yang et al. [15] investigated the quasi in situ compression recovery properties of NiTi alloys with the L-DED process. Results showed that the material can be completely recovered under 4% deformation and the B19′ martensite phase content and dislocation density are basically unchanged. However, the recovery rate was only 90% and the unrecoverable strain was 0.86% at 8% deformation.

Li et al. [16] reviewed the literature on the in situ detection, generation, effects and countermeasures of spatter in L-PBF, which has an intrinsic correlation with the forming quality. Although many researchers have provided insights into the melt pool, microstructure and mechanical property, reviews of spatter in L-PBF are still limited. Hence, this work is expected to pave the way towards a novel generation of highly efficient and intelligent L-PBF systems.

## 4. Applications of Laser Additive Manufacturing in This Special Issue

The findings of this Special Issue article are expected to provide guidance and reference for scientists and engineers in the fields of aerospace (e.g., copper/steel multi-material engine thrust chambers [7], titanium alloy lightweight structural components [6,12]), biology (e.g., Mg–Zn [3], Fe/ZnS bone-repair material [11]), energy (e.g., neutron tube shells [8]) and micromachines (e.g., programmable micro-robots [5]).

Tao et al. [5] demonstrated a novel femtosecond LAM process with smart materials for soft interactive hydrogel micro-machines. These programmable stimuli-responsive matrices mechanized hydrogels into robotic applications at the micro/nanoscale (<300 × 300 × 100 μm^3^). Benefiting from high-efficiency two-photon polymerization (TPP), nanometer feature size (<200 nm) and flexible digitalized modeling technique, many micro/nanoscale hydrogel robots or machines have become obtainable with respect to future interdisciplinary applications.

Wang et al. [6] investigated the influence of process parameters on the densification, microstructure and mechanical properties of a Ti–6Al–4V alloy printed by L-PBF, followed by annealing treatment. The results show that the microstructure can be tailored by altering the scanning speed and annealing temperature. The maximum elongation of 14% can be achieved at an annealing temperature of 950 °C, which was 79% higher than that of as-printed samples. Meanwhile, ultimate tensile strength larger than 1000 MPa can be maintained, which still meets the application requirements of the forged Ti–6Al–4V alloy.

Zhou et al. [11] introduced zinc sulfide (ZnS) into Fe bone-implants manufactured using the LAM technique, which promoted the collapse of the passive film and accelerated the degradation rate of the Fe matrix. This work indicated that the Fe/ZnS biocomposite is able to act as a promising candidate for bone-repair material.

To conclude, we would like to acknowledge all the authors for their contributions, adding to the success of this Special Issue in *Micromachines* as well as the reviewers whose feedback helped to improve the quality of the published papers. 

## References

[1] Chen K., Wan H., Fang X., Chen H. (2022). Laser Additive Manufacturing of Anti-Tetrachiral Endovascular Stents with Negative Poisson’s Ratio and Favorable Cytocompatibility. Micromachines.

[2] Song C., Hu Z., Xiao Y., Li Y., Yang Y. (2022). Study on Interfacial Bonding Properties of NiTi/CuSn10 Dissimilar Materials by Selective Laser Melting. Micromachines.

[3] Yang Y., Wang W., Yang M., Yang Y., Wang D., Liu Z., Shuai C. (2021). Laser-Sintered Mg-Zn Supersaturated Solid Solution with High Corrosion Resistance. Micromachines.

[4] Zheng X., Meng J., Liu Y. (2022). Strain Rate Dependence of Compressive Mechanical Properties of Polyamide and Its Composite Fabricated Using Selective Laser Sintering under Saturated-Water Conditions. Micromachines.

[5] Tao Y., Lu C., Deng C., Long J., Ren Y., Dai Z., Tong Z., Wang X., Meng S., Zhang W. (2022). Four-Dimensional Stimuli-Responsive Hydrogels Micro-Structured via Femtosecond Laser Additive Manufacturing. Micromachines.

[6] Wang D., Wang H., Chen X., Liu Y., Lu D., Liu X., Han C. (2022). Densification, Tailored Microstructure, and Mechanical Properties of Selective Laser Melted Ti–6Al–4V Alloy via Annealing Heat Treatment. Micromachines.

[7] Zhang W., Zhang B., Xiao H., Yang H., Wang Y., Zhu H. (2021). A Layer-Dependent Analytical Model for Printability Assessment of Additive Manufacturing Copper/Steel Multi-Material Components by Directed Energy Deposition. Micromachines.

[8] Kang M., Qiang Y., Zhu C., Xiang X., Zhou D., Peng Z., Xie X., Zhu Q. (2022). Hybrid Dissection for Neutron Tube Shell via Continuous-Wave Laser and Ultra-Short Pulse Laser. Micromachines.

[9] Meng L., Long J., Yang H., Shen W., Li C., Yang C., Wang M., Li J. (2022). Femtosecond Laser Treatment for Improving the Corrosion Resistance of Selective Laser Melted 17-4PH Stainless Steel. Micromachines.

[10] Wang X., Jiang J., Tian Y. (2022). A Review on Macroscopic and Microstructural Features of Metallic Coating Created by Pulsed Laser Material Deposition. Micromachines.

[11] Zhou Y., Xu L., Yang Y., Wang J., Wang D., Shen L. (2022). Microstructure and Corrosion Behavior of Iron Based Biocomposites Prepared by Laser Additive Manufacturing. Micromachines.

[12] Cui Y., Cai J., Li Z., Jiao Z., Hu L., Hu J. (2022). Effect of Porosity on Dynamic Response of Additive Manufacturing Ti-6Al-4V Alloys. Micromachines.

[13] Xu Z., Wang D., Song W., Tang C., Sun P., Yang J., Hu Q., Zeng X. (2022). Microstructure and Wear of W-Particle-Reinforced Al Alloys Prepared by Laser Melt Injection. Micromachines.

[14] Zhang L., Li Y., Li S., Gong P., Chen Q., Geng H., Sun M., Sun Q., Hao L. (2022). Fabrication of Titanium and Copper-Coated Diamond/Copper Composites via Selective Laser Melting. Micromachines.

[15] Yang X., Wang S., Pan H., Zhang C., Chen J., Zhang X., Gao L. (2022). Microstructure Transformation in Laser Additive Manufactured NiTi Alloy with Quasi-In-Situ Compression. Micromachines.

[16] Li Z., Li H., Yin J., Li Y., Nie Z., Li X., You D., Guan K., Duan W., Cao L. (2022). Review of Spatter in Laser Powder Bed Fusion Additive Manufacturing: In Situ Detection, Generation, Effects, and Countermeasures. Micromachines.

